# Article Watch

**DOI:** 10.7171/001c.160040

**Published:** 2026-04-17

**Authors:** Clive A Slaughter

**Affiliations:** 1 Basic Sciences AU-UGA Medical Partnership

**Keywords:** Article Watch

## Abstract

This column highlights recently published articles that are of interest to the readership of this publication. We encourage ABRF members to forward information on articles they feel are important and useful to Clive Slaughter, AU-UGA Medical Partnership, 1425 Prince Avenue, Athens GA 30606. Tel; (706) 713-2216: Fax; (706) 713-2221: Email; cslaught@uga.edu or to any member of the editorial board. Article summaries reflect the reviewer’s opinions and not necessarily those of the Association.

## NUCLEIC ACIDS SQUENCING & GENOTYPING

Neville MDC, Lawson ARJ, Sanghvi R, Abascal F, Pham MH, Cagan A, Nicola PA, Bayzetinova T, Baez-Ortega A, Roberts K, Lensing SV, Widaa S, Alcantara RE, García MP, Wadge S, Stratton MR, Campbell PJ, Small K, Martincorena I, Hurles ME, Rahbari R. Sperm sequencing reveals extensive positive selection in the male germline. *Nature* 647;2025:421-428. doi: 10.1038/s41586-025-09448-3

Calvet F, Blanco Martinez-Illescas R, Muiños F, Tretiakova M, Latorre-Esteves ES, Fredrickson J, Andrianova M, Pellegrini S, Huber A R, Ramis-Zaldivar JE, An S C, Thieme E, Kohrn BF, Grau ML, Gonzalez-Perez A, Lopez-Bigas N, Risques RA. Sex and smoking bias in the selection of somatic mutations in human bladder. *Nature* 647;2025:436-444. doi: 10.1038/s41586-025-09521-x

Lawson ARJ, Abascal F, Nicola PA, Lensing SV, Roberts AL, Kalantzis G, Baez-Ortega A, Brzozowska N, El-Sayed Moustafa JS, Vaitkute D, Jakupovic B, Nessa A, Wadge S, Österdahl MF, Paterson AL, Rassl DM, Alcantara RE, O’Neill L, Widaa S, Austin-Guest S, Neville MDC, Przybilla MJ, Cheng W, Morra M, Sykes L, Mayho M, Müller-Sienerth N, Williams N, Alexander D, Harvey LMR, Clarke T, Byrne A, Blundell JR, Young MD, Mahbubani KTA, Saeb-Parsy K, Martin HC, Stratton MR, Campbell PJ, Rahbari R, Small KS, Martincorena I. Somatic mutation and selection at population scale. *Nature* 647;2025:411-420. doi: 10.1038/s41586-025-09584-w

Somatic mutation in human tissues is widespread and common. It occurs in both rapidly dividing and nondividing cells throughout life and is of acknowledged clinical interest. Of special concern is whether mutation at particular genes may confer selective advantage within populations of dividing cells (i.e., mediates clonal expansion). Because individual mutations often occur in single cells or small clones, study of the phenomenon requires single-molecule sequencing. However, it also requires a sequencing error rate that is much lower than the mutation rate. Abascal F. et al. (*Nature* 593;2021:405-410) previously showed that these conditions may be met by a duplex sequencing protocol, called NanoSeq, that provides an error rate of five errors per billion base pairs, two orders of magnitude lower than the mutation rate in single cells from mammalian tissues. A number of groups are now adopting or improving this technology to characterize somatic mutation in diverse inquiries. Neville et al. study sperm samples from men aged 24–75 years. Mutations in sperm may be transmitted to children and could account for increased incidence of certain congenital disorders with increasing paternal age. Neville et al. identify 35,000 germline coding mutations, including ones in 40 genes that provide evidence for positive selection. Calvet et al. seek to explain the higher rate of bladder cancer among men and smokers. They perform deep sequencing of 16 targeted genes in some 400,000 haploid genomes from 79 normal bladder samples derived from 45 people. They identify increased rates of mutation in the telomerase gene *TERT* promoter with age and smoking, and increased rates of truncating mutation in three other genes among men. Lawson et al. study 1042 samples of normal oral epithelium and 371 blood samples. They detect 46 genes under positive selection in oral epithelium and over 62,000 driver mutations, as well as some genes under negative selection that indicate their essential nature. These studies illustrate the rising importance of the study of somatic mutation enabled by sequencing at very low error rates.

## MACROMOLECULAR CHARACTERIZATION

Straub M S, Harder O F, Mowry N J, Barrass S V, Hruby J, Drabbels M, Lorenz U J. Laser flash melting cryo-EM samples to overcome preferred orientation. *Nature Methods* 22;2025:1880-1886. doi: 10.1038/s41592-025-02796-y

Cryo-electron microscopy (cryo-EM) has become a widely used approach in determining the structure of biological macromolecules. However, sample preparation in this methodology can sometimes be problematic, particularly when macromolecules adopt a preferred orientation at the interface between the cryo-EM specimen support and the water in which these macromolecules were dissolved prior to its vitrification. Such preferred orientation may limit resolution, or even preclude 3D reconstruction. Straub et al. here build upon the group’s earlier observation that flash melting and revitrification of the sample may alter the orientation of some large, highly symmetric proteins, presumably by dissociating them from the air-water interface and retrapping them before they can reconnect with it. In the present work, the authors systematically explore the conditions under which flash melting and revitrification are performed, including the shape and duration of laser pulses employed for melting. They also test the effect of applying a layer of amorphous ice to the sample prior to revitrification. Varying these conditions is found to populate previously disfavored orientations for a diverse set of proteins. The results help identify the competing processes that govern the behavior of macromolecules during vitrification. They suggest ways in which alteration of the support surface or sample may be combined with flash melting and revitrification to optimize results for particular macromolecular species, and provide methods that may be implemented with existing equipment readily modified for the purpose.

Kirschbaum C, Bennett JL, Robinson CV. Deep structural characterization of protein-bound lipids via native MS and ultraviolet photodissociation. *Analytical Chemistry* 97;2025:19331-19339. doi: 10.1021/acs.analchem.5c03691

The function of proteins may be strongly affected by the identity of lipids with which they interact. The repertoire of lipids available for interaction with membrane proteins changes as proteins move from one subcellular compartment to another, and they may also change with alterations in physiological or pathological conditions. Analysis of the lipids interacting with a particular protein may be accomplished by extracting the lipids from a lipid-protein complex, followed by separate lipid analysis. However, distinguishing the lipids directly bound to the protein from the ensemble of lipids present with it may be difficult in this way. Kirschbaum et al. describe methodology based upon native mass spectrometry combined with ultraviolet photodissociation (UVPD) to resolve such uncertainty. They first acquire a mass spectrum of the protein under conditions that preserve its native interaction with lipids. For membrane proteins that lack a simple 1:1 interaction with a specific lipid in a defined binding pocket, but bind a variety of lipid species with variable stoichiometry at a hydrophobic surface, the authors quantify the individual lipids in an MS^1^ spectrum by MS^2^-guided curve fitting of the partially resolved MS^1^ species. They then isolate the ensemble of protein-lipid complexes for collision-induced dissociation (CID) or higher-energy collision-induced dissociation (HCD) to release the lipids as neutral molecules or charged ions. The protein moieties are then detected as charge-reduced ions. In this way, they purify true protein-lipid complexes in the gas phase. The lipids are isolated in an ion trap and subjected to CID to determine the lipid class and acyl chain composition. The authors deploy 213 nm UVPD to determine acyl chain structure (e.g., C=C bond positions, cyclopropane rings) of phospholipids by examining informative fragments in MS^3^ spectra. They determine acyl chain positions on the glycerol backbone by CID of sodiated ions to generate dioxolane fragments, which yield position-specific cross-ring fragments upon UVPD for detection in MS^4^ spectra. For analysis of proteins from which lipids have been stripped by detergent purification, the proteins may be incubated with a lipid extract, and the resulting complexes analyzed in a similar way. The entire procedure is conducted without any requirement for prior derivatization of sample.

## MASS SPECTROMETRY

Nesbitt DJ, Mertz KL, Probasco MD, Peters-Clarke TM, Oman TJ, Syka JEP, Quarmby ST, Coon JJ. Implementation of infrared-activated negative electron transfer dissociation (IR-NETD) using xenon on a quadrupole-Orbitrap-quadrupole linear ion trap mass spectrometer. *Journal of the American Society for Mass Spectrometry* 37;2026:301-309. doi: 10.1021/jasms.5c00345

Nesbitt et al. describe instrument modifications and methodology for improved electron transfer dissociation (ETD) in the negative ion mode, which is especially useful for analysis of nucleic acids, oligosaccharides, and acidic proteins. The authors supply infrared (IR) photons to provide supplemental activation energy for analyte precursor ions during the ETD ion-ion fragmentation process to counteract the tendency for precursor ions of low charge state to remain undissociated owing to noncovalent interactions between the resulting product ions. The result is improved efficiency of fragment ion production. The use of photons for this purpose avoids the hydrogen rearrangements and altered isotopic distributions that occur when collisional or electron-based dissociation are employed. The authors also replace the fluoranthene gas conventionally used as the ETD reagent gas with xenon as a reagent for ion-ion fragmentation. Xenon imparts more energy when reacting with precursor anions, generating more neutral loss fragments; xenon is also easily introduced into the system. To implement IR photodissociation, Nesbitt et al. attach an IR laser to the Orbitrap Ascend system from Thermo Fisher Scientific (San Jose, CA, USA). This is a quadrupole-Orbitrap-quadrupole linear ion trap system. They add a Peltier device to help align the laser coaxially with the optics of the instrument. The authors demonstrate the utility of the system by analyzing a synthetic 21-mer small interfering RNA molecule with complex modifications.

Rider RL, Hampton J, Xi Z, Lantz C, Yun SD, Liu W, Viner R, Laganowsky A, Russell DH. Dissecting heterogeneous populations of protein-complex samples using direct mass technology. *Analytical Chemistry* 97;2025:27057-27063. doi: 10.1021/acs.analchem.5c05771

Charge detection mass spectrometry (CDMS), a technique in which the mass of a particle is deduced by independent measurement of both *m/z* and charge state (*z*) of individual ions oscillating within an electrostatic linear ion trap, has proven useful for measuring the mass of very large particles that would yield conventional electrospray mass spectra so congested as to preclude interpretation. The technique has been made widely available by implementation in Orbitrap mass spectrometers. Rider et al. here demonstrate extension of CDMS application to a quantitative analysis of equilibria between interconverting subforms of a protein of <100 kDa. The subject of their study is the transthyretin (TTR) tetramer. The authors resolve and quantify TTR homotetramers and the various heterotetramer species formed between native TTR subunits and TTR subunits bearing a C-terminal extension of nine amino acids. They also resolve and quantify the binding of thyroxine to these various species. The results show that the C-terminal extension accelerates subunit dissociation and enhances thyroxine binding affinity, thereby defining the effect of the C-terminus, which resides at the dimer-dimer interface, on the properties of the molecule.

Krupa S, Szuberla W, Nizioł J, Ossolińska A, Ossoliński K, Ruman T. Broadband collision-induced dissociation mass spectrometry imaging. *Journal of the American Society for Mass Spectrometry* 36;2025:1443-1455. doi: 10.1021/jasms.5c00045

Krupa et al. explore a novel combination of existing mass spectral techniques to test a new biological imaging modality that promises significant advantages for imaging of small molecules such as lipids, metabolites, and drug molecules. They employ an infrared (IR) laser of millijoule (mJ) energy level and 2.94 µm wavelength for desorption of molecules from frozen tissue sections. This IR energy couples with O–H stretching of hydrogen-bonded water, and is suitable for any hydrated biological material. It penetrates more deeply into tissues than other desorption methods, such as secondary ion mass spectrometry (SIMS), desorption electrospray ionization (DESI), or ultraviolet laser desorption in matrix-assisted laser desorption/ionization (MALDI). The increased sampling depth thus enhances ion yields. It also avoids the need for application of a matrix as required by MALDI. The imaging sample chamber is pressurized with nitrogen, which sweeps the plume of desorbed material through a tube into an electrospray ionization source. The methodology is named laser ablation remote electrospray ionization (LARESI), a gentle ionization protocol that produces predominantly intact molecular ions. In the present work, it is coupled with broadband collision-induced dissociation (bbCID) for MS/MS analysis of molecular structure. The methodology is implemented in this study with a modified Impact II mass spectrometer from Bruker Corp., but it is generally compatible with other instrument systems. The authors demonstrate the capabilities of the methodology in studies of lipid and metabolite distribution in clinical tissue samples of human bladder and kidney cancers.

## METABOLOMICS

Bansal S, Gori M, Quaye J A, Gadda G, Wang B. Detection and analysis of reactive oxygen species (ROS): buffer components are not bystanders. *Analytical Chemistry* 97;2025:14931-14942. doi: 10.1021/acs.analchem.4c07070

The development of a plethora of clinical conditions is trustingly attributed to reactive oxygen species (ROS), but the reactivity of these species makes them exceedingly difficult to detect and quantify because they are short-lived and probably low in effective concentration. The effects of antioxidants are correspondingly hard to delineate. Bansal et al. here highlight the effects of experimental conditions on the measurement of ROS by demonstrating the chemical reactivity of commonly used buffers toward them. The authors show that HEPES, Tris, MES, citrate, ammonium acetate, and phosphate-buffered saline all consume sodium hypochlorite rapidly. These buffer components also interact with boronates, which are commonly used in the measurement of hydrogen peroxide. The authors document false negative results in the detection of peroxynitrite when using a boronate-based probe. The results with these two species, the most abundant ROS in biological systems, exemplify the kinds of effects investigators may anticipate when designing experimental studies for investigation of reactive species.

Lai Y, Xu A, Dong L, Li S, Li Y, Liu J. Quantitative tracking of nanoplastic uptake and distribution in Zebrafish by single-particle inductively coupled plasma mass spectrometry. *Analytical Chemistry* 97;2025:27298-27307. doi: 10.1021/acs.analchem.5c05336

Nanoplastic particles have attracted much concern due to their environmental prevalence and potential toxicity. The distribution of nanoplastics in tissues of experimentally exposed organisms is commonly studied with fluorescently labeled nanoplastic species, but leaching of fluorophores and high cellular autofluorescence lead to concern about the reliability of results. The present article exemplifies increasing interest in the search for mass spectrometric methods to study the fate of nanoplastics within exposed organisms. The present authors approach the problem by deploying inductively coupled plasma mass spectrometry to detect nanoparticles labeled with a heavy metal, europium (Eu). Zebrafish exposed for seven days to polystyrene nanoparticles doped with an Eu chelate are dissected, and nanoparticles are extracted from the tissues by digestion of the tissue matrix with a strongly alkaline aqueous solution of tetramethylammonium hydroxide. This treatment successfully avoids leaching of Eu. The results reveal the largest accumulation in the intestine, and, interestingly, the presence of some in the brain, which suggests a breach of the blood-brain barrier. Also of interest, no particles are detected in eggs, suggesting resistance to penetration through the chorion.

McCarty KD, Guengerich FP. Mass spectral analysis of sterols and other steroids in different ionization modes: sensitivity and oxidation artifacts. *Journal of the American Society for Mass Spectrometry* 36;202:1702-1717. doi: 10.1021/jasms.5c00099

Electrospray ionization (ESI) and, subsequently, atmospheric pressure chemical ionization (APCI) have contributed importantly to the mass spectrometric analysis of steroids, which is an application of clinical utility. APCI, often the more sensitive method for ionization of such neutral molecules, unfortunately results in a variety of artifactual modifications that arise during the analysis. McCarty et al. here provide details of one family of such modifications — the loss of 2, 4, or even 6 amu — from positively charged steroid molecular ions, MH^+^, during APCI, which causes significant confusion during structural assignment. The authors systematically compare detection sensitivity for various steroid families achieved with ESI and APCI and with ESI using a heated electrospray source with a vaporizer maintained at 300 ˚C. They also test the addition of NH_4_F to the mobile phase during ESI. They determine that the losses of 2*n* amu are largely the result of oxidation at carbinol (C–OH) groups. The data are useful in the design of LC-MS conditions for the analysis of steroids and sterols.

## PROTEOMICS

Genovese BN, Randhawa N, Grigorean G, Drazenovich T, Phinney BS, Van Rompay KK A, Yee J, Mazet JAK, Bird BH. Specimen inactivation methods for proteomics ─ comparisons of irradiation, chemical, and heat treatments on downstream serum analyses. *Journal of Proteome Research* 24;2025:5282-5291. doi: 10.1021/acs.jproteome.5c00290

Genovese et al. compare methods for inactivation of pathogens for the purpose of safety in proteomic studies of serum. They find that heating samples at 95 ˚C for 5 min, although low-cost and easy to implement, produces the most serious effects on sample integrity in terms of protein/peptide yield and introduction of artifactual chemical modifications. Chemical chaotropic agents such as TRIzol (a mixture of phenol, guanidine isothiocyanate, ammonium thiocyanate, sodium acetate, and glycerol) are advantageous for yield but require special precautions both for reproducible sample handling and for chemical safety. Heating at 56 ˚C for 1 h provides convenience and high yield for proteins but poor yield for peptides. The authors favor γ-irradiation if available. It provides effective neutralization of pathogens, the best reproducibility of proteomic results and the least negative effect on protein/peptide yield.

Phlairaharn T, Shannon A E, Zeng X, Truong D-J J, Schoof E M, Ye Z, Searle B C. Improving proteomic dynamic range with multiple accumulation precursor mass spectrometry. *Journal of Proteome Research* 24;2025:5116-5126. doi: 10.1021/acs.jproteome.5c00469

In a typical data-dependent proteomic analysis of a peptide mixture in an Orbitrap mass spectrometer, acquisition of a precursor ion spectrum in a given duty cycle takes much less time than the subsequent acquisition of the product ion spectrum from the selected precursor. During product ion scanning, precursor analysis is on hold. In the present article, Phlairaharn et al. exploit this unused time for improved precursor scanning. They reason that the dynamic range of precursor scans is limited by the most abundant ions within the scanned *m*/*z* interval. To detect larger numbers of precursors within this interval, they split it up into subintervals scanned serially across the entire range of *m*/*z* values in the time available within the duty cycle. This approach is analogous to the “boxcar” acquisition method that was developed originally to improve dynamic range at the expense of not collecting product ion spectra. The authors achieve a nearly 2x improvement in precursor dynamic range. The methodology can be implemented using existing hardware and software tools. Although implemented in this article for proteomic analysis, the method can be deployed to improve precursor detection in the identification and quantification of molecular species comprising any complex mixture by tandem mass spectrometry in an Orbitrap instrument.

Huang T, Wang J, Stukalov A, Kreimer S, Zhao X, Cantrell LS, Zhou X, Brewer A, Ho G, Murolo F, Quach K, Figa M, Just S, Castro G, Elgierari E, Benz R W, Motamedchaboki K, Batzoglou S, Farokhzad OC, Van Eyk JE, Siddiqui A. Multiplexed nanoparticle protein corona enables accurate and precise deep plasma proteomics. *Journal of Proteome Research* 24;2025:5884-5893. doi: 10.1021/acs.jproteome.5c00729

Here is a benchmark study for the proteomic capabilities of the nanoparticle corona system provided by Seer, Inc. (Redwood City, CA, USA). The corona methodology seeks to ameliorate the analytic challenge of global identification and quantitation of the proteins in mixtures such as plasma, the components of which occur over an enormous range of abundance. Nanoparticles with varying surface coatings are used to bind proteins with varying selectivity dependent upon protein characteristics such as size, shape, charge, and functional groups. Proteins with high abundance but low affinity for a particular nanoparticle are supposed to be displaced by low abundance proteins with a higher affinity for that particle. A suitable choice of particle selectivities therefore favors detection of rare proteins that would otherwise be missed, in effect expanding the dynamic range of the analytical system. Fold-difference in the abundance of particular proteins between samples is amenable to direct measurement of ion intensities, provided the relative affinities of the component proteins is preserved. The present article documents the fold-change accuracy, linearity, and precision with which plasma proteins can be measured across the required dynamic range. Performance is assessed by a multiple spike-in method. Varying quantities of bovine plasma are spiked into a constant background of human plasma covering bovine:human ratios ranging from 1:1 to 1:127, and the results are compared with a neat bovine plasma preparation omitting corona selection. Using an Orbitrap Astral mass spectrometer system from Thermo Fisher Scientific (Waltham, MA, USA), the authors identify 7,459 bovine plasma proteins (57,206 peptides) across all dilutions, whereas only 1,644 proteins (11,421 peptides) are identified in neat plasma. Subtracting proteins/peptides shared between bovine and human leaves 2,664 bovine proteins (4,511 bovine peptides). (The authors do also detect 323 bovine proteins in pure human samples, possibly due to carryover in the analytical system.) Fold-change accuracy is preserved throughout the dilution series, demonstrating no effect of proteome background on accuracy.

## FUNCTIONAL GENOMICS & PROTEOMICS

Chauhan VP, Sharp PA, Langer R. Engineered prime editors with minimal genomic errors. *Nature* 646;2025:1254-1260. doi: 10.1038/s41586-025-09537-3

In base editing, a Cas9 nickase that complexes with DNA at a position determined in a sequence-dependent manner by a guide RNA (gRNA) is fused to a deaminase enzyme that alters a specific base nearby, causing a single base replacement upon DNA replication or repair. In prime editing, however, the Cas9 nickase is fused to a reverse transcriptase. The guide RNA determining the target is also extended to encode the intended edit, which may be a nucleotide replacement or an indel. The strand bearing the 3′ end of the nick anneals with the guide RNA, and the reverse transcriptase installs the desired sequence as an extension of this 3′ end. But this requires the prime editing guide RNA (pegRNA) to displace the strand on the 5′ side of the nick – a disfavored event. Chauhan et al. improve the efficiency of prime editing by engineering the Cas9 nickase to facilitate displacement of this 5′ strand. They do so by relaxing the positioning of the nick, thereby promoting degradation of the 5′ strand. Diminished competition with the 5′ strand produces a striking reduction in the frequency with which editing errors occur due to unintended introduction of indels. Editing efficiency improves several-fold. The suppression of these errors improves the potential utility of prime editing for therapeutic purposes and facilitates the interpretation of results from pooled screening to assess the functional consequences of multiple mutations of a protein of interest. The authors’ observations also help explain large variations in efficiency of editing when producing different mutations in the same target protein.

Gould SI, Wuest AN, Dong K, Johnson GA, Hsu A, Narendra VK, Atwa O, Levine SS, Liu DR, Sánchez Rivera FJ. High-throughput evaluation of genetic variants with prime editing sensor libraries. *Nature Biotechnology* 43;2025:1648-1662. doi: 10.1038/s41587-024-02172-9

For any particular gene of interest, there is an enduring need for high-throughput methods by which to assess the repertoire of mutations (single nucleotide variants or small indels) that will produce disease-causing changes in the structure/function of the encoded protein. Screening systems based on overexpression of protein variants have long provided a solution to this problem, but they entail expression of variants at supraphysiologic levels that may alter the biological impact of the protein in such a way as to produce false positive or false negative results. Prime editing has the potential to circumvent this difficulty because it provides a means of expressing large numbers of variants in pooled screens under conditions more closely approximating those in vivo. The difficulty with this approach is that different pegRNAs may induce editing with different efficiencies, making quantitative assessment of their effects difficult to assess in pooled screens. Gould et al. address this problem by designing a sensor methodology that measures the efficiency of each edit. As sensors, they employ artificial copies of the endogenous target sites. These enable systematic empirical assessment of the efficiency of individual pegRNAs and, in turn, leads to an improved tool for the design of prime editing libraries, which the authors make freely available. In this study, the methodology is deployed to assess the effects of mutations in the transcription factor TP53. The clinical importance of the study derives from the *TP53* gene being the most frequently mutated in cancer. The authors screen over 1,000 *TP53* variants that have been associated with cancer. The results will help to resolve questions surrounding the biological effects of these variants, notably the functional impact of reduction in active TP53 levels in heterozygotes and the occurrence among them of neomorphic, gain-of-function variants.

Lindenhofer D, Bauman JR, Hawkins JA, Fitzgerald D, Yildiz U, Jung H, Korosteleva A, Marttinen M, Kueblbeck M, Zaugg JB, Noh K-M, Dietrich S, Huber W, Stegle O, Steinmetz LM. Functional phenotyping of genomic variants using joint multiomic single-cell DNA–RNA sequencing. *Nature Methods* 22;2025:2032-2041. doi: 10.1038/s41592-025-02805-0

Lindenhofer et al. address the limitations imposed in pooled screens due to variable efficiency of genome editing by linking genomic DNA sequencing for detection of genomic DNA (gDNA) with RNA sequencing to determine resulting effects on gene expression in a single-cell assay. Cells are fixed and permeabilized, and reverse transcription is performed with custom primers for a panel of up to 480 selected RNA targets. Cells containing complementary DNA (cDNA) and gDNA are analyzed using the Tapestri workflow from Mission Bio (San Francisco, CA, USA). The authors are able to assess the effect of directed gene variants, both coding and noncoding, and, in the same cells, to determine the zygosity of the variants induced. The capability to extend the analysis to noncoding variants is important because noncoding mutations are prominent among disease-causing genetic changes. The authors study B cell lymphomas and are able to associate B cell receptor signaling with mutation burden. They anticipate that the methodology will assist analysis of intratumor heterogeneity and cancer evolution.

Chao Y-K, Wu M, Gong Q, Chen F. A genetically encoded device for transcriptome storage in mammalian cells. *Science* 392;2026:eadz9353. doi: 10.1126/science.adz9353

Seeking a way to achieve transcriptome-wide capture of mRNA produced by living cells over a period of days, in order to track gene activity during extended periods of physiological change, Chao et al. engineer particles known as vaults to accomplish such capture. Vaults are ribonucleoprotein complexes of enigmatic function. They weigh 13 MDa and are widely expressed at high levels in the cytoplasm of eukaryotic cells. Chao et al. fuse poly(A)-binding proteins (PABPs), which bind mRNAs *via* their poly(A) tails, to the major vault proteins (MVPs) that comprise the outer shell of the vault complex. The resulting engineered vault complex binds and stores mRNAs in a nuclease resistant form within the complex. Inducible expression of the engineered vault protein thus allows analysis of the transcriptome in a cell lineage within a defined time window that is terminated when the vault particles are recovered. Chao et al. show that the mRNAs captured by the vault provide a faithful representation of the transcriptome in the host cell. They can retrieve mRNAs over a period of seven days. And the engineered vault doesn’t perturb normal cell physiology. The authors illustrate application of the vault technology by recording stress responses to heat shock and hypoxia, and characterize the response of cancer cells upon initiation of targeted therapy that results in a drug-insensitive “persister” state in some cancer cells. The vault technology offers a new way to accomplish transcriptome-wide molecular recording in mammalian cells.

Musunuru K, Grandinette SA, Wang X, Hudson TR, Briseno K, Berry AM, Hacker JL, Hsu A, Silverstein RA, Hille LT, Ogul AN, Robinson-Garvin NA, Small JC, McCague S, Burke SM, Wright CM, Bick S, Indurthi V, Sharma S, Jepperson M, Vakulskas CA, Collingwood M, Keogh K, Jacobi A, Sturgeon M, Brommel C, Schmaljohn E, Kurgan G, Osborne T, Zhang H, Kinney K, Rettig G, Barbosa CJ, Semple SC, Tam YK, Lutz C, George LA, Kleinstiver BP, Liu DR, Ng K, Kassim SH, Giannikopoulos P, Alameh M-G, Urnov FD, Ahrens-Nicklas RC. Patient-specific in vivo gene editing to treat a rare genetic disease. *New England Journal of Medicine* 392;2025:2235-2243. doi: 10.1056/NEJMoa2504747

Most pathogenic genetic variants are individually rare, but collectively they affect surprisingly large numbers of individuals. Development of therapies to treat children affected by uncommon variants is impeded by the tremendous investment of resources required. Musunuru et al. here document the development of personalized base-editing therapy for a single patient with neonatal-onset carbamoyl-phosphate synthase 1 (CPS1) deficiency, a very rare urea cycle defect with a mortality rate of 50% in early infancy. The story spans an eight-month period after the patient’s birth. One month after the patient’s birth, cassettes for both the patient’s *CPS1* alleles were synthesized and transduced into cultured cells with a lentiviral vector. Two months after the patient’s birth, adenine base editors were screened and a guide RNA (gRNA) targeting one of the alleles were tested in culture. Toxicity testing in cynomolgus monkeys and in vivo efficacy testing in a mouse model followed. A clinical batch of lipid nanoparticles was produced five months after the patient’s birth and tested in cultured cells. After prophylactic immunosuppression to avoid an immune response to the CPS1 protein, the first of two infusions of the nanoparticle vector was administered to the patient 208 days after birth. The patient received a second dose 22 days later because of incomplete biochemical correction. The second dose resulted in his tolerance of an increased level of dietary protein. Dosing with nitrogen-scavenging medication could be reduced to one-half of the starting dose. Lipid nanoparticle delivery allows readministration, which is contraindicated with adeno-associated virus vectors because of their immunogenicity. This study exemplifies therapies that have now been developed for a large number of hepatic inborn errors of metabolism. The platform described in the present example can be replicated for further patients by using the same lipid nanoparticle and mRNA but with gRNA customized for the patient-specific variant. The present study represents a prototype for future rapid development of personalized gene-editing therapies for additional genetic diseases.

## CELL BIOLOGY

Du W, Nair PR, Forjaz A, Phillip JM, Wu P-H, Wirtz D. Selecting the optimal cell migration assay: fundamentals and practical guidelines. *Nature Methods* 23;2026:30-42. doi: 10.1038/s41592-025-02890-1

Wu P-H, Phillip JM, Du W, Forjaz A, Nair PR, Wirtz D. Methods to analyze cell migration data: fundamentals and practical guidelines. *Nature Methods* 23;2026:43-55. doi: 10.1038/s41592-025-02935-5

A pair of review articles provides guidance on ways to measure cell migration and analyze the data acquired. Cell movement is a fundamental cellular process that is as important for understanding the normal and pathological behavior of cells and tissues as proliferation or differentiation. Yet, there is little standardization of the choice of methods for the study of cell migration or criteria for evaluation of results. Cells use distinct modes and mechanisms of migration depending on their state of differentiation and their biological context, including presence of neighboring cell types, the nature of the extracellular matrix, and the presence of signaling molecules. A large number of in vitro assays have been developed, and some are available commercially. They mimic to various degrees the conditions and circumstances encountered by particular cells in vivo. The authors of the present articles describe 10 commercial or custom cell migration assays for use in vitro and 2 assays for use in vivo. Du et al. describe information acquired in each type of assay, convenience of implementation, cost, commercial source if any, number of conditions that can be probed, the need for microscopy, provision for performing molecular analyses after the assay, capability to tack different cell types simultaneously, capability to track single cells, what in vivo processes the assay mimics, and the number of cells needed for measurement. Wu et al. provide guidelines for assay-type selection and describe how quantitative cell migration parameters are calculated from the raw data. Different types of migration assays are often used interchangeably, but it is hoped that the present work will increase the stringency of experimental design in the field and stimulate further development of the technology presently available.

Kudo T, Meireles AM, Moncada R, Chen Y, Wu P, Gould J, Hu X, Kornfeld O, Jesudason R, Foo C, Höckendorf B, Corrada Bravo H, Town JP, Wei R, Rios A, Chandrasekar V, Heinlein M, Chuong AS, Cai S, Lu CS, Coelho P, Mis M, Celen C, Kljavin N, Jiang J, Richmond D, Thakore P, Benito-Gutiérrez E, Geiger-Schuller K, Hleap JS, Kayagaki N, de Sousa e Melo F, McGinnis L, Li B, Singh A, Garraway L, Rozenblatt-Rosen O, Regev A, Lubeck E. Multiplexed, image-based pooled screens in primary cells and tissues with PerturbView. *Nature Biotechnology* 43;2025:1091-1100. doi: 10.1038/s41587-024-02391-0

Gu J, Iyer A, Wesley B, Taglialatela A, Leuzzi G, Hangai S, Decker A, Gu R, Klickstein N, Shuai Y, Jankovic K, Parker-Burns L, Jin Y, Zhang JY, Hong J, Niu X, Costa J A, Pezet M G, Chou J, Chen CC, Paiva M, Snoeck H-W, Landau DA, Azizi E, Chan E M, Ciccia A, Gaublomme JT. Mapping multimodal phenotypes to perturbations in cells and tissue with CRISPRmap. *Nature Biotechnology* 43;2025:1101-1115. doi: 10.1038/s41587-024-02386-x

Fandrey C I, Jentzsch M, Konopka P, Hoch A, Blumenstock K, Zackria A, Maasewerd S, Lovotti M, Lapp D J, Gohr FN, Suwara P, Świeżewski J, Rossnagel L, Gobs F, Cristodaro M, Muhandes L, Behrendt R, Lam MC, Walgenbach KJ, Bald T, Schmidt F I, Latz E, Schmid-Burgk JL. NIS-Seq enables cell-type-agnostic optical perturbation screening. *Nature Biotechnology* 43;2025:1337-1347. doi: 10.1038/s41587-024-02516-5

Three groups make substantial contributions to optical pooled screening (OPS) methodology. In OPS, CRISPR genetic perturbations are induced in cells in a high-throughput pooled screen. The phenotype each cell expresses is assessed with the help of optical microscopy and is linked to the genetic perturbation the cell bears, which is assessed by in situ genotyping using sequencing-by-synthesis. All three groups address the problem that the perturbation barcodes used to identify the alteration in individual cells are expressed at low levels in many cell types. Kudo et al. extend the so-called zombie protocol, in which an RNA barcode is transcribed in vitro in fixed cells by T7 bacteriophage RNA polymerase. This involves adding a T7 promoter for barcode expression alongside the CRISPR gRNA promoter responsible for inducing the genetic perturbation. Fandrey et al. adopt a similar approach using T7 in vitro transcription. Gu et al. abandon sequencing-by-synthesis in favor of identifying the barcode by multiplexed immunofluorescence. This accomplishes RNA identification by combinatorial hybridization with fluorophore-labeled DNA oligonucleotides. The improved barcode detection afforded by all three groups generalizes the methodology to application in diverse cell types.

## IMAGING

Duswald K, Pichler V, Kopatz V, Limberger T, Karl V, Hennerbichler D, Zimmerleiter R, Wadsak W, Hettich M, Gruber ES, Kenner L, Brandstetter M. Detection of unlabeled polystyrene micro- and nanoplastics in mammalian tissue by optical photothermal infrared spectroscopy. *Analytical Chemistry* 97;2025:16714-16722. doi: 10.1021/acs.analchem.4c05400

Fourier transform infrared spectroscopy (FTIR) and Raman spectroscopy have become commonly used methods for detecting micro- and nanoplastic particles in tissues, but these methods impose limitations either with regard to particle size or fluorescence. The present study explores the use of optical photothermal IR spectroscopy (O-PTIR) for the purpose. In this technique, the sample absorbs energy from an infrared laser. Resultant localized heating causes expansion that is measured using visible light optics. This process allows acquisition of IR spectra beyond the IR diffraction limit. It therefore enables chemical analysis at the submicron scale. Using spherical polystyrene particles, the present authors demonstrate identification and localization of particles that are 200 nm in diameter in formalin-fixed, paraffin-embedded mouse kidney tissue sections.

Nowakowska AM, Pieczara A, Korona W, Borek-Dorosz A, Adamczyk A, Dawiec P, Leszczenko P, Orleanska J, Machalska E, Orzechowska B, Orzechowska S, Brzozowski K, Krynicka W, Majzner K, Malek K, Baranska M. The Raman map of the human cell. *Analytical Chemistry* 97;2025:16374-16382. doi: 10.1021/acs.analchem.5c02035

Raman spectroscopy provides nondestructive methods for analysis of structure and molecular composition of living cells and tissues. It can provide a “chemical fingerprint,” valuable in distinguishing normal from pathologic states and also valuable in monitoring responses to stimuli in real time. Raman confocal microscopy is an imaging technique capable of submicron spatial resolution and may therefore be used to map subcellular structures. Nowakowska et al. provide a detailed Raman map of one selected cell type. They document Raman signatures of the nucleus, nucleoli, the endoplasmic reticulum-rich perinuclear region, mitochondria, lipid droplets, and cytoplasm, and then compare the detected features with the spectra of biological reference compounds. The work is performed with an Alpha 300 confocal Raman microscope from WITec (Ulm, Germany), a member of the Oxford Instruments Group. The authors study a human aortic endothelial cell line. The resulting Raman map of a cell will be useful as a reference standard for future investigation of the utility of Raman imaging in both the research and clinical arenas.

## MACROMOLECULAR SYNTHESIS

Yin Y, Arneson R, Yuan Y, Fang S. Long oligos: direct chemical synthesis of genes with up to 1728 nucleotides. *Chemical Science* 16;2025:1966-1973. doi: 10.1039/d4sc06958g

Zhang M, Hu Y, Huang H, Shi Y. Chemically synthesized ultra-long DNA as building blocks to accelerate complex gene construction in synthetic biology. *bioRxiv* 2025:2025.2004.2002.646740. doi: 10.1101/2025.04.02.646740

Phosphoramidite chemistry is generally thought to be limited to the synthesis of oligonucleotides that are ∼200 nucleotides in length. Yin et al., however, demonstrate chemical synthesis of an 800-mer green fluorescent protein gene and a 1728-mer ϕ29 DNA polymerase gene using this methodology. They deploy two innovations to achieve these results. Firstly, they perform the synthesis on glass beads with a smooth surface instead of controlled-pore glass beads. This change improves the efficiency of the coupling reaction, which they attribute to the elimination of steric hindrance within the pores of porous glass. The efficiency of the capping reaction is apparently also improved: the present syntheses achieve error rates of 0.043%, an improvement of >10x compared with literature values of 0.58% in the synthesis of 200-mers, despite the increased length of oligonucleotides synthesized in the present work. Secondly, the authors utilize a method they previously devised for purification of the final full-length oligonucleotide product. Termed “catching-by-polymerization,” it involves tagging the full-length product with a polymerizable phosphoramidite. The tagged molecule is then conjugated to a polyacrylamide gel. Failure sequences are capped and therefore do not become tagged. Failure sequences are hence removed during washing of the acrylamide gel prior to cleavage of the final product from the gel. Zhang et al. also employ nonporous glass for extended chemical synthesis. They employ biotinylated primers complementary to the full-length product for purification. Enzymatic methods have also become available for the synthesis of long oligonucleotides, but the chemical methods are particularly advantageous for the synthesis of long oligonucleotides with certain difficult sequences.

## DESIGN & DEVELOPMENT OF THERAPEUTICS

Hunter TL, Bao Y, Zhang Y, Matsuda D, Riener R, Wang A, Li JJ, Soldevila F, Chu DSH, Nguyen DP, Yong Q-C, Ross B, Nguyen M, Vestal J, Roberts S, Galvan D, Vega JB, Jhung D, Butcher M, Nguyen J, Zhang S, Fernandez C, Chen J, Herrera C, Kuo Y, Pica E M, Mondal G, Mammen AL, Scholler J, Tanis SP, Sievers SA, Frantz AM, Adams GB, Shawver L, Farzaneh-Far R, Rosenzweig M, Karmali PP, Bot AI, June CH, Aghajanian H. In vivo CAR T cell generation to treat cancer and autoimmune disease. *Science* 388;2025:1311-1317. doi: 10.1126/science.ads8473

Widespread interest exists in exploration of autologous chimeric antigen receptor (CAR) T cells and NK cells for treatment of disease. An expanding range of relatively common diseases is being studied, which now includes solid tumors and autoimmune conditions in addition to approved methods for leukemias/lymphomas and multiple myeloma. Linked to this interest is a focus on development of methods for CAR cell induction in vivo to overcome the large investment of resources required to create CAR cells ex vivo. The cost and complexity of ex vivo production presently limits accessibility of this treatment modality. Hunter et al. here demonstrate in vivo production of CAR T cells using targeted lipid nanoparticles. The composition of the nanoparticles incorporates a cationic lipid to reduce uptake by the liver, and a suitable protein is incorporated to target the nanoparticles to CD8^+^ T cells. The mRNA payload encodes an anti-CD19 CAR designed to enable the engineered T cells to deplete B lymphocytes. The authors report control of a B cell-like tumor engrafted into humanized mice, and the rapid, profound depletion of B cells in cynomolgus monkeys. When B cells repopulate following treatment, they are predominantly naïve, indicating a favorable immune reset. The methodology provides advantages over the use of integrating viral vectors for in vivo CAR T cell therapy. It avoids long-term depletion of target cells and the risk of forming T cell lymphomas. The success of this study encourages optimism to proceed with testing for safety and feasibility for therapeutic application.

